# Salvianolic acid B attenuates inflammation and prevent pathologic fibrosis by inhibiting CD36-mediated activation of the PI3K-Akt signaling pathway in frozen shoulder

**DOI:** 10.3389/fphar.2023.1230174

**Published:** 2023-08-01

**Authors:** Yan Yan, Min Zhou, Ke Meng, Chuanhai Zhou, Xiaoyu Jia, Xinhao Li, Dedong Cui, Menglei Yu, Yiyong Tang, Ming Li, Jinming Zhang, Zhuo Wang, Jingyi Hou, Rui Yang

**Affiliations:** ^1^ Department of Orthopedics, Sun Yat-sen Memorial Hospital, Sun Yat-sen University, Guangzhou, China; ^2^ Department of Orthopedics, The Eighth Affiliated Hospital, Sun Yat-sen University, Shenzhen, China; ^3^ Sun Yat-sen University, Guangzhou, China

**Keywords:** frozen shoulder, fibrosis, CD36, salvianolic acid B, inflammatory cytokines, PI3K-Akt signaling pathway

## Abstract

Frozen shoulder (FS) is characterized by pain and limited range of motion (ROM). Inflammation and fibrosis are accepted as main pathologic processes associated with the development of FS. However, the intrinsic mechanisms underlying pathologic fibrosis remain unclear. We aimed to elucidate the key molecules involved in pathologic fibrosis and explore new therapeutic targets for FS. Synovial fibroblasts isolated from patient biopsies were identified using immunofluorescence. Western blotting, RT-qPCR, cell adhesion tests, and would-healing assays were used to evaluate the fibrosis-related functions of synovial fibroblasts. Elevated cluster of differentiation 36 (*CD36*) expression was detected in FS using Western blotting and immunohistochemistry. Salvianolic acid b (SaB) inhibited CD36, blocking synovial fibroblast-induced inflammation and fibrosis. Our RNA-seq data showed that knocking down CD36 dramatically impaired the capacity of synovial fibroblasts for cell adhesion and that the PI3K-Akt signaling pathway may be crucial to the fibrotic process of FS. By up-regulating *CD36* and inhibiting the phosphorylation of Akt, we demonstrated that CD36 promotes pathologic fibrosis by activating the PI3k-Akt pathway. Finally, rats treated with SaB had improved ROM and less collagen fiber deposition than the FS model group.

**Conclusion:** SaB attenuates inflammation and inhibited the CD36-mediated activation of the PI3K-Akt signaling pathway to block pathologic fibrosis of FS in *vitro* and *in vivo* models.

## 1 Introduction

Frozen shoulder (FS) or “adhesive capsulitis” is characterized by progressively worsening shoulder pain and reduced range of motion (ROM) (Neviaser and Neviaser, 2011; Mezian et al., 2022). It is a common orthopedic problem with an incidence of 2%–5% and an average acute phase of 1–2.5 years (Shah and Lewis, 2007). Currently, physiotherapy and intra-articular glucocorticoid injections are the mainstay treatments for FS (Ramirez, 2019). However, the majority of patients endure prolonged shoulder pain and restricted joint movement due to the slow therapeutic onset of physiotherapy and the short-term effects of glucocorticoids (Cho et al., 2019). Approximately 40% of FS patients experience partial alleviation after the initial phase but continue to experience ongoing low-level restriction and pain (Shaffer et al., 1992; Neviaser and Neviaser, 2011). Although many theories explain the onset of FS, its specific pathogenesis remains unclear. Typically, inflammation and pathologic fibrosis in the shoulder joint are thought to be the main etiological processes in FS, and pathologic fibrosis is intrinsically linked to restricted ROM (Tamai et al., 2014). As a result, numerous research efforts have honed in on identifying the mechanisms responsible for the pathologic fibrosis in FS (Blessing et al., 2019).

In 1997, Rodeo et al. reported that capsular fibrosis could be induced by persistently stimuling with TGF-β1 and PDGF (Rodeo et al., 1997). Later, it was found that inflammatory cytokines, including IL-6, IL-8, TNF-α, and M-CSF, were associated with the FS fibrotic process (Kabbabe et al., 2010). Additional studies reported that the balance between MMPs/TIMPs and markers of stromal fibroblast activation (PDPN, VCAM, MCAM) influences the excessive accumulation of extracellular matrix (ECM) in FS (Rodeo et al., 1997; Lubis and Lubis, 2013; Akbar et al., 2019; Dakin et al., 2019). Lubis et al. reported that FS patients exhibit a lower MMP/TIMP ratio compared to controls, contributing to capsular fibrosis (Lubis and Lubis, 2013). Furthermore, Hagiwara et al. conducted gene chip research on FS capsule tissues showing elevated gene expression in fibrosis, inflammation, and cartilage formation, suggesting that all three mechanisms might be crucial in the development of FS (Hagiwara et al., 2012). These studies, however, were primarily concerned with the development of pathologic fibrosis at the tissue level and were unable to fully explain the cellular changes in the capsule.

Fibroblasts are believed to be responsible for many fibrotic diseases including FS (Guyot et al., 2006; Sun et al., 2016; Tsoyi et al., 2018; Yang et al., 2022). There is a general consensus that with persistent inflammation, fibroblasts continue to transform into α-SMA (+) myofibroblasts and secrete a large amount of ECM, which ultimately triggers pathologic fibrosis (Hinz et al., 2007). In the shoulder joint, fibroblasts are also referred to as “synovial fibroblasts” or “fibroblast-like synoviocytes” (Bunker and Anthony, 1995; Ha et al., 2014). Hettrich et al. reported that the FS capsule shows a higher prevalence of myofibroblasts compared to control capsules, suggesting that synovial fibroblasts may hasten the development of pathologic fibrosis in FS (Hettrich et al., 2016). At the same time, Akbar et al. demonstrated that synovial fibroblasts are activated in FS and produce more inflammatory cytokines such as IL-6, IL-8, and CCL-20; these findings further elucidate the synergistic effects between activated synovial fibroblasts and dysregulated inflammatory cytokines (Akbar et al., 2019). In addition, Akbar et al. found that IL-17A can induce profibrotic and inflammatory responses in fibroblasts through the TRAF-6/NF-*κ*B pathway in FS (Akbar et al., 2021). However, further research is still needed to determine the precise connection between synovial fibroblasts and the pathologic fibrosis of FS as well as its underlying mechanisms.

Recently, the cluster of differentiation 36 (CD36) was reported to play a potentially important role in fibrotic diseases (Chen et al., 2009; Souza et al., 2016; Lin et al., 2019; Liu et al., 2021). CD36 is widely expressed in many cell types such as monocytes, macrophages, platelets, endothelial cells, fibroblasts, adipocytes, and some epithelial cells (Pepino et al., 2014). As a cell membrane receptor, CD36 contributes to several physiological and pathologic processes by interacting with other membrane and cytoplasmic proteins (Silverstein and Febbraio, 2009; Wang and Li, 2019). CD36 takes part in the activation of latent TGF-*β*1 after binding to TSP-1 on the membrane of alveolar epithelial cells, which further contributes to the deposition of ECM and the progression of pulmonary fibrosis (Chen et al., 2009). By influencing lipid metabolism, CD36 can accelerate the development of chronic inflammation and fibrosis in nonalcoholic fatty liver disease; microRNA-29 can halt these effects by suppressing the expression of *CD36* (Lin et al., 2019). This is supported by renal disease; patients with proteinuria have higher levels of *CD36* expression in the renal proximal convoluted tubule, while treatment of mice with induced chronic kidney disease with the CD36 inhibitor 5A peptide reduces interstitial fibrosis (Souza et al., 2016). JUN initiates fibrosis by regulating *CD36* in human fibroblasts and mouse wounds and *CD36* deletion can prevent or even reverse JUN-mediated fibrosis (Liu et al., 2021). Thus, in this study, we hypothesized that CD36 expressed on synovial fibroblasts participates in cell adhesion, ECM deposition, and fibrosis and contributes to the development of FS since pathologic fibrosis in the shoulder capsule parallels that seen in equivalent liver, lung, and kidney disorders.

Salvianolic acid B (SaB) is a small water-soluble molecule isolated from the root of *Salviae Miltiorrhizae*, and was identified as an effective CD36 antagonist in a recent high-throughput screen (Bao et al., 2012). *Salviae miltiorrhizae* is widely used in the treatment of stable angina pectoris in traditional Chinese medicine because it promotes blood circulation (Du et al., 2020). SaB may also show anti-fibrotic, anti-inflammatory, anti-oxidant, and anti-apoptotic properties (Xiao et al., 2020). SaB inhibits the expression of some inflammatory factors and collagen proteins such as COL 1 and COL 3 in some fibrotic diseases, including cirrhosis and lung fibrosis (Tao et al., 2021; Zhang et al., 2021). SaB was also shown to inhibit TGF-β1 expression in hepatic stellate cells, thus antagonizing the activation of the TGF-β1/Smad pathway and reducing the deposition of collagen fibers in the liver (Wu et al., 2019). In addition, SaB hinders pathologic skin fibrosis in diseases such as systemic sclerosis and hyperplastic scars (Wu et al., 2019; Liu et al., 2021). However, the relationship between SaB and CD36 in FS remains to be elucidated.

Our main aim in this study was to characterize the expression of *CD36* in human FS capsule tissue in comparison to a healthy control group and elucidate CD36 signaling in synovial fibroblasts to determine if targeting CD36 may offer a novel therapeutic approach in fibrosis.

## 2 Material and methods

### 2.1 Study approval

The current study was approved by the Ethics Committee of the Sun Yat-sen Memorial Hospital of Sun Yat-sen University, Guangzhou, China. Full informed consent was obtained from all patients. The sample size for tissue- and cell-based assays were determined based on sample availability and technical needs.

### 2.2 Patients and controls

Patients with frozen shoulder were diagnosed according to the medical history, physical examination and arthroscopic manifestations; patients with recurrent dislocation of the shoulder and no limitation of passive ROM were selected as the control group (Hagiwara et al., 2012). We use 3.5 mm grasping biopsy forceps to obtain synovial tissue biopsies under arthroscopy. 12 patients with FS and 12 patients with habitual dislocation of the shoulder from October 2021 to June 2022 in Sun Yat-sen University were recruited consecutively. All patients signed the contract for permission to use their tissue for experimental purposes. Demographics and characteristics of patients in both groups are shown in Table 1.

**TABLE 1:** Baseline characteristics.

Outcome	FS	Control	*p*-value
Number	12	12	
Age (years)	57.88	51.11	0.065
Sex			
Male (N)	6	8	
Female (N)	6	8	
ROM (passive)			
Flex	93.18 ± 6.25	154.39 ± 9.65	<0.001 *
Abd	73.69 ± 6.98	149.37 ± 12.48	<0.001 *
ER	16.34 ± 3.31	59.68 ± 3.97	<0.001 *
Shoulder function			
ASES	40.36 ± 8.34	68.32 ± 6.21	0.019 *
Constant Murley	43.21 ± 7.11	69.46 ± 5.47	0.011 *
UCLA	14.97 ± 2.91	23.61 ± 2.55	0.059

Mean values are expressed as mean ± standard deviation *Statistical significance (a = 0.05).

ROM: range of motion; Flex: flexion; Abd: abduction; ER: external rotation; ASES: american shoulder and elbow surgeons scale; UCLA: the university of California at Los Angeles shoulder rating scale.

### 2.3 Cell isolation and culture

The biopsied samples were rinsed adequately three times with phosphate buffered saline (PBS; Jetbio, China). Thereafter, the samples were minced finely and digested with 0.2% type I collagenase (Sigma Aldrich, Germany) in high-glucose Dulbecco’s modified Eagle’s medium (high-glucose DMEM; Gibco, United States) containing 10% fetal bovine serum (FBS; Gibco, United States). After being incubated for 2 h at 37°C, cells were collected through centrifugation (1,000 rpm, 5 min), washed three times with PBS, and resuspended with high-glucose DMEM containing 10% FBS. After then, cells were transferred to a T25 culture flask (Jetbio, China) and allowed to attach for 3–5 days. Those non-adherent cells were removed after changing the medium. The medium was changed every 2–3 days. The synovial fibroblasts were passaged when reaching 80%–90% confluence, and those from passage three to five were used for subsequent experiments.

### 2.4 Identification of synovial fibroblasts

When the fibroblast cultures reached 80%–90% confluence, the adherent cells were digested (in 0.05% trypsin-0.02% EDTA; Gibco, United States) and seeded in a 6-well plate with pre-set culture slides (1.5 × 10^5 cells/well). Fibroblasts were identified by immunofluorescence staining with CD68 and vimentin antibodies. Briefly, fibroblasts were cultured on culture slides, washed with PBS, and fixed in 4% paraformaldehyde (Sigma Aldrich, Germany) at room temperature for 20 min. Subsequently, the cells were permeabilized with 0.5% Triton X-100 (Sigma Aldrich, Germany) at room temperature for 15 min and blocked in 10% goat serum (BOSTER, China) at room temperature for 1 h, followed by incubation with primary antibodies against vimentin (1:50; BOSTER, China) or CD68 (1:100; Abcam, United Kingdom) overnight at 4°C. The next day, the slides were incubated with Alexa Fluor 488-conjugated secondary antibody (goat anti-rabbit; 1:1,000; Beyotime, China) at room temperature for 1 h and washed three times with PBS for 5 min. The cells were incubated with DAPI (1:1,000; Beyotime, China) at room temperature for 5 min to label the nuclei, and the stained slides were examined under a fluorescence microscope (Olympus Corporation, Japan) at ×20 magnification.

### 2.5 Quantitative reverse transcription polymerase chain reaction (RT-qPCR)

Total RNA of the synovial fibroblasts was isolated using a RNA Purification Kit (EZBioscience, United States) and transcribed into cDNA with a PrimeScript RT reagent kit (Accurate Biology, China). RT-qPCR analyses were then performed using a LightCycler 480 RealTime PCR System (Roche, Basel, Switzerland) with a SYBR Premix Ex Taq (Accurate Biology, China). The results were normalized to the expression level of GAPDH and the relative expression of each gene was determined with the 2^(−ΔΔCt) method. The forward and reverse primers for each gene are listed in Table 2.

**TABLE 2:** Forward and reverse primers for each gene.

Gene	Forward	Reverse
*CD36*	5′-CTT​TGG​CTT​AAT​GAG​ACT​GGG​AC-3′	5′-GCA​ACA​AAC​ATC​ACC​ACA​CCA-3′
COL1	5′-GAG​GGC​CAA​GAC​GAA​GAC​ATC-3′	5′-CAG​ATC​ACG​TCA​TCG​CAC​AAC-3′
COL3	5′-GCC​AAA​TAT​GTG​TCT​GTG​ACT​CA-3′	5′-GGG​CGA​GTA​GGA​GCA​GTT​G-3′
α-SMA	5′-CTA​TGA​GGG​CTA​TGC​CTT​GCC-3′	5′-GCT​CAG​CAG​TAG​TAA​CGA​AGG​A-3′
FN	5′-CGG​TGG​CTG​TCA​GTC​AAA​G-3′	5′-AAA​CCT​CGG​CTT​CCT​CCA​TAA-3′
TNF-α	5′-GAG​GCC​AAG​CCC​TGG​TAT​G-3′	5′-CGG​GCC​GAT​TGA​TCT​CAG​C-3′
TGF-β1	5′-GGC​CAG​ATC​CTG​TCC​AAG​C-3′	5′-GTG​GGT​TTC​CAC​CAT​TAG​CAC-3′
IL-1β	5′-ATG​ATG​GCT​TAT​TAC​AGT​GGC​AA-3′	5′-GTC​GGA​GAT​TCG​TAG​CTG​GA-3′
IL-6	5′-ACT​CAC​CTC​TTC​AGA​ACG​AAT​TG-3′	5′-CCA​TCT​TTG​GAA​GGT​TCA​GGT​TG-3′
CXCL-10	5′-GTG​GCA​TTC​AAG​GAG​TAC​CTC-3′	5′-TGA​TGG​CCT​TCG​ATT​CTG​GAT​T-3′
GAPDH	5′-GGA​GCG​AGA​TCC​CTC​CAA​AAT-3′	5′-GGC​TGT​TGT​CAT​ACT​TCT​CAT​GG-3′

*CD36*: cluster of differentiation 36; COL, 1: collagen type I; COL, 3: collagen type III; α-SMA: α-Smooth Muscle Actin; FN: fibronectin; TNF-α: tumor necrosis factor-α; TGF-β1: transforming growth factor beta 1; IL-1β: interleukin 1β; IL-6: interleukin 6; CXCL-10: C-X-C Motif Chemokine Ligand 10; GAPDH: glyceraldehyde-3-phosphate dehydrogenase.

### 2.6 Enzyme-linked immunosorbent assays (ELISAs)

After administration of SaB for 48 h, concentrations of TNF-α, TGF-β1, IL-1β, IL-6 and CXCL-10 in synovial fibroblasts culture supernatant were detected using Quantikine ELISA kits for TNF-α, TGF-β1, IL-1β, IL-6 and CXCL-10 (R&D Systems, United States) according to the manufacturer’s protocol.

### 2.7 Western blotting

Synovial fibroblasts were lysed using cell lysis buffer (CWBiotech, China) containing Protease inhibitors (1:100; CWBiotech, China) and Phosphatase inhibitors (1:100; CWBiotech, China) and the protein concentrations were quantified with a protein assay kit (Vazyme, China). Equal amounts of protein extracts were electro-phoresed on 8 or 10% gels and then transferred to PVDF membranes (Millipore, United States). The PVDF membranes were blocked with 5% bovine serum albumin (Biosharp, China) and incubated with primary antibodies against GAPDH (Abcam, United Kingdom), COL 1 (Abcam, United Kingdom), COL 3 (Abcam, United Kingdom), FN (Abcam, United Kingdom), *α*-SMA (Abcam, United Kingdom), CD36 (Abcam, United Kingdom), PI3K (Cell Signaling Technology, United States), p-Akt (Cell Signaling Technology, United States), Akt (Cell Signaling Technology, United States), p-Smad 2/3 (Cell Signaling Technology, United States), Smad 2/3(Cell Signaling Technology, United States), p-p38(Cell Signaling Technology, United States), p38 (Cell Signaling Technology, United States), ERK1/2 (Servicebio, China), p-ERK1/2 (Servicebio, China), Wnt-10b (Abcam, United Kingdom), β-Catenin (Abcam, United Kingdom), p-β-Catenin (Abcam, United Kingdom) overnight at 4°C. The PVDF membranes were then incubated with appropriate secondary antibodies (diluted 1:5000; ABclonal, China). The specific antibody-antigen complexes were detected using an enhanced chemiluminescence kit (Vazyme, China). Band images were captured using the Bio-Rad Gel Doc XR documentation system (Bio-Rad, United States). Relative protein expression levels were determined by ImageJ (version 1.6; National Institutes of Health, United States) and standardized to GAPDH.

### 2.8 Histology and immunohistochemistry

Part of the excised synovial samples of every patient were immediately fixed in 10% formalin for 16–24 h. The samples were then embedded in paraffin wax and sectioned into 4–6 µm for further hematoxylin and eosin (H&E) staining, Masson’s trichrome staining, and Safranin O-Fast Green staining (Solarbio, China) according to the manufacturer’s protocol.

Immunohistochemistry (IHC): After deparaffinization and dehydration, the sections underwent antigen retrieval in citrate buffer, quenched in 3% H_2_O_2_, and blocked with goat serum. The sections were further incubated with the specific antibody against COL 1 (Abcam, United Kingdom) and COL 3 (Abcam, United Kingdom) overnight at 4°*C. SP* Rabbit and Mouse HRP Kit (DAB) (CW Biotech, China) were used to detect specific labeling according to the manufacturer’s protocol. Hematoxylin (Solarbio, China) was used for counterstain sections.

### 2.9 Cell counting Kit-8 test

Cell Counting Kit-8 (CCK-8, APExBIO, United States) was applied to determine the cytotoxicity of salvianolic acid B (SaB, Sigma Aldrich, Germany). Synovial fibroblasts from the frozen shoulder group were seeded onto 96-well plates at a density of 3,000 cells per well and cultured with various concentrations of SaB. Also implanted and grown without SaB were synovial fibroblasts from the healthy control group. Subsequently, 10 uL of the CCK-8 reagent was added to each well at 24 h or 48 h, or 72 h after seeding and used to quantify the cell number after incubating for 1 h at 37°C. All experiments were performed in triplicate. The absorbance at an OD of 450 nm was measured using a UV-spectrophotometer (Life Technologies, United States).

### 2.10 Cell adhesion test

Cell adhesion ability was determined by cell-substrate adhesion assays as previously described (Yang et al., 2020). Briefly, 100 µL of the adhesion substrate fibronectin (10 μg/mL; Sigma Aldrich, Germany) was added to flat bottom 96-well plates and incubated for 60 min at ambient temperature. Three wells were left blank to determine the background binding of crystal violet. Synovial fibroblasts from the frozen shoulder and healthy control group were pretreated according to different experimental conditions in 6-well plates and were subsequently harvested with trypsin and resuspended at 2.85 * 10^5/mL. Then, 50 μL cell suspension was added to 50 µL PBS prior to the addition of this cell suspension into the prepared wells. Synovial fibroblasts were allowed to adhere for 20 min at 37°C in a 5% CO_2_ incubator. Nonadherent cells were removed by tapping the plate gently and washing the wells twice with 100 µL PBS. Attached cells were fixed by using 100 µL of 5% glutaraldehyde (Sigma Aldrich, Germany) and subsequently stained by adding 100 µL of 0.1% crystal violet (Beyotime, China). Before dissolving the crystal violet, the cell adhesion morphology was captured by an inverted microscope (Nikon, Japan). The crystal violet dye was solubilized in 100 µL of 10% acetic acid and the absorbance was measured at 570 nm using a UV-spectrophotometer (Life Technologies, United States). All experiments were performed in triplicate. The normalized absorbance was obtained by subtracting the background from the blank wells.

### 2.11 Wound-healing assay

Synovial fibroblasts treated according to different experimental conditions were seeded in a 6-well plate (1.5 × 10^5 cells/well) and cultured to confluence. A horizontal artificial wound was introduced with a P-200 pipette tip in each well. The data for the wounded area was recorded at 0 h, 24 h, and 48 h with a microscope. All experiments were performed in triplicate.

### 2.12 Cell transfection

Si*CD36* and scrambled negative control siRNA were obtained from Genepharma Co. Ltd. (Suzhou, China). The *CD36* overexpression plasmid and negative control were obtained from Genecreate Co. Ltd. (Wuhan, China). The final concentration of siRNA was 20 μM, and the final plasmid concentration was 100 μg/μL. Synovial fibroblasts were seeded in 6-well plates (1.5 * 10^5 cells/well) and transfected with siRNA, *CD36* overexpression plasmid, or negative controls via Lipofectamine 3,000 (Thermo Fisher Scientific, United States) according to the manufacturer’s instructions. After 48 h, cells were harvested to quantify the mRNA or protein expression.

### 2.13 RNA-seq and analysis

Total mRNA extracted from the synovium tissue or the synovial fibroblasts was purified and reversely transcribed into cDNA. The amplified cDNA was then sequenced on Illumina Novaseq 6000 platform (Illumina, United States) by LC science (Hangzhou, China), following the vendor’s recommended protocols. Genes differential expression analysis was performed by DESeq2 software between two different groups (and by edgeR between two samples). The genes with the parameter of false discovery rate (FDR) below 0.05 and absolute fold change ≥2 were considered differentially expressed genes. Differentially expressed genes were then subjected to enrichment analysis of Gene ontology (GO) functions and Kyoto Encyclopedia of Genes and Genomes (KEGG) pathways.

### 2.14 Animal model setup

Eighteen 12-week-old female Sprague-Dawley rats (200–300 g) were randomly divided into Group A: control group (*n* = 6), Group B: FS model group (*n* = 6) and Group C: SaB intervention group (*n* = 6). In the FS model group and SaB intervention group, rats were immobilized with molding plaster for 3 weeks to induce the FS. Immobilization procedures were conducted under intraperitoneal anesthesia using pentobarbital (30 mg/kg). Immobilization was successfully accomplished by applying molding plaster around the entire left arm, including the shoulder and thorax (Kim et al., 2016). The shoulder was fully adducted and internally rotated, and the forearm was fastened against the chest wall. Adjust the tightness of the molding plaster to ensure the rats were able to breathe, self-feed, walk, and survive for over 3 weeks (Supplementary Figure S2). In the SaB intervention group, we applied SaB to rats by intraperitoneal injection (50 mg/kg), while an equal volume of PBS was used in the other two groups. During the whole research, no complications were observed following the immobilization. 3 weeks later, molding plaster was removed. The rats were used for gait analysis and then euthanized for measurement of the abduction angle and further histologic evaluation of the capsule.

### 2.15 Gait analysis

Gait analysis was performed by measuring the stride length of both sides between the front and rear paws on a grid paper (Kim et al., 2021). The left-side paws of all rats were dabbed with a red-color inkpad, and the right-side paws were dabbed with a blue-color inkpad. Rats were made to walk on a grid paper by luring them with food. To make the rats walk straight, both sides of the grid paper were blocked with a wall. The distances between the front and rear paws on both sides were measured, and the longest distance of the measurement was defined as the stride length. This measurement was repeated 5 times in all rats, and the average was used for the analysis.

### 2.16 X-ray examination

To verify the effectiveness of model-setting, X-rays films of rats were acquired before euthanasia. Rats were positioned prone on a scanning table with tape just below the X-ray source under general anesthesia. The neutral and abduction positions’ X-ray films were collected using a flatbed scanner. First, the rats’ fore claws were naturally placed on both sides of the body, and the routine X-ray examination was performed to produce the neutral position X-ray film. Then fix the rats’ fore claws with the No. Four silk threads, while another end of which was tied to a 10 g weight. Place the rats prone on the X-ray inspection bed, fix both limbs in the abducted posture with the 10 g weights suspended from both sides of the bed, and perform routine X-ray examination (Supplementary Figure S3). Consider the axis of the humerus and the lateral edge of the scapula as the two sides of an angle with the humeral head’s center as the vertex. The calculation of angle was finished by using the system’s own software and repeated by the same radiologist for three times, the average value of which was taken for further statistical analysis.

### 2.17 ROM measure

Each rat was euthanized under sufficient intraperitoneal anesthesia using pentobarbital (200 mg/kg) and, and the whole limbs from the scapula of whom were harvested *en bloc*. During the harvest, the origin and insertion of all muscles affecting the glenohumeral joint motion (deltoid, supraspinatus, infraspinatus, subscapularis, biceps, and triceps) were preserved, and extra care was taken not to damage the glenohumeral joint capsule. The scapula was fixed using two 27-G injection needles through the superior and inferior angles of the scapula on a Styrofoam block, thereby eliminating scapular motion. Further, the points on the distal end of the humerus when the shoulder was maximally adducted and maximally abducted were marked with 27-G injection needles. The angle between the scapular medial border and humerus shaft was measured using a protractor, and the glenohumeral ROM was calculated by subtracting the angle in a maximally adducted position from the angle in a maximally abducted position 40. To avoid dehydration of soft tissue on the joint and to maintain reproducibility of each experiment, the whole procedure was performed within 15 min from the tissue harvest.

### 2.18 Statistical analysis

Statistical analysis was performed with SPSS 23.0 software (Chicago, IL, United States). All data are presented as means ± standard deviations (SD). Student’s t-test was performed to assess differences between two groups. *p* values <0.05 were considered statistically significant.

## 3 Results

### 3.1 Identification of synovial fibroblasts

**FIGURE 1:**
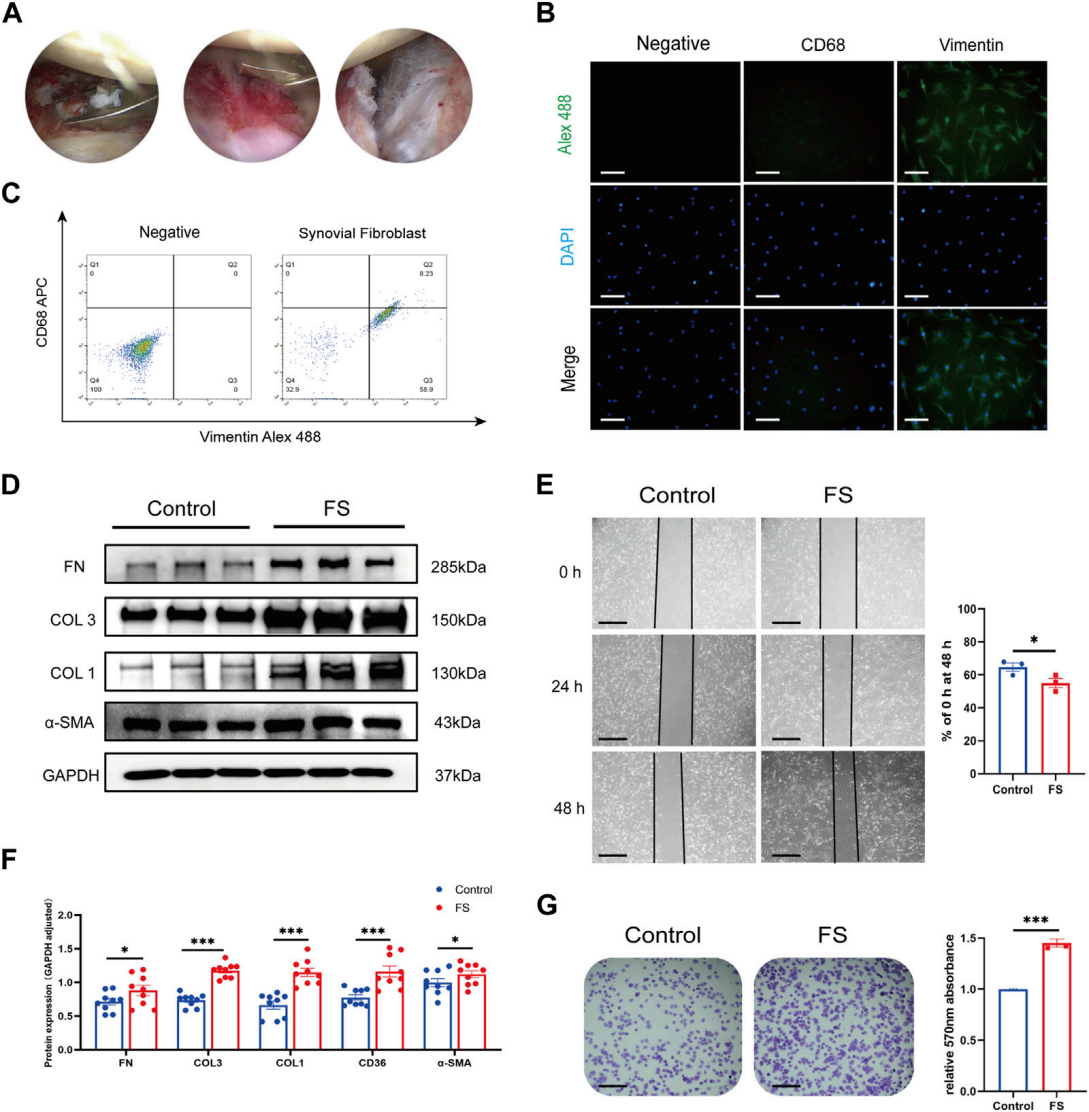
Extraction and identification of synovial fibroblasts and evaluation of fibrosis-related abilities. **(A)** Arthroscopic view of the right shoulder in a 68-year-old woman with primary frozen shoulder. Biopsies were obtained from inflammatory synovium and thickened capsule with a punch forceps. **(B,C)** Cells extracted from synovium tissue expressed the specific markers for fibroblasts with positive vimentin while negative CD68. Scale bar: 25 µm. **(D,F)** Western blotting results showed that synovial fibroblasts in FS expressed more COL 1, COL 3, FN, and *α*-SMA than that in the control group. **(E,G)** Cell adhesion test and Wound-healing showed that synovial fibroblasts from FS were associated with the enhanced fibrosis-related ability. Scale bar: 50 μm *p < 0.05; **p < 0.01; ***p < 0.001.

The synovial fibroblasts used in this study were isolated and cultured from the synovium of the human shoulder and passaged to the 3-fifth generation. Inflamed synovium and thickened joint capsules were seen in FS patients during arthroscopy (Figure 1A). We further identified the phenotype of fibroblasts through immunofluorescence. Cells from both the FS and control groups expressed the specific markers for fibroblasts with positive vimentin and were negative for the macrophage marker CD68 (Figures 1B, C). Our Western blotting analysis showed that the synovial fibroblasts from the FS group expressed greater COL 1, COL 3, FN, and *α*-SMA compared to controls. Finally, the cell adhesion test and wound-healing assay confirmed our previous conclusion that synovial fibroblasts from the FS group were associated with an enhanced ability to generate fibrosis (Figures 1D–G).

### 3.2 *CD36* expression in FS and control groups

**FIGURE 2:**
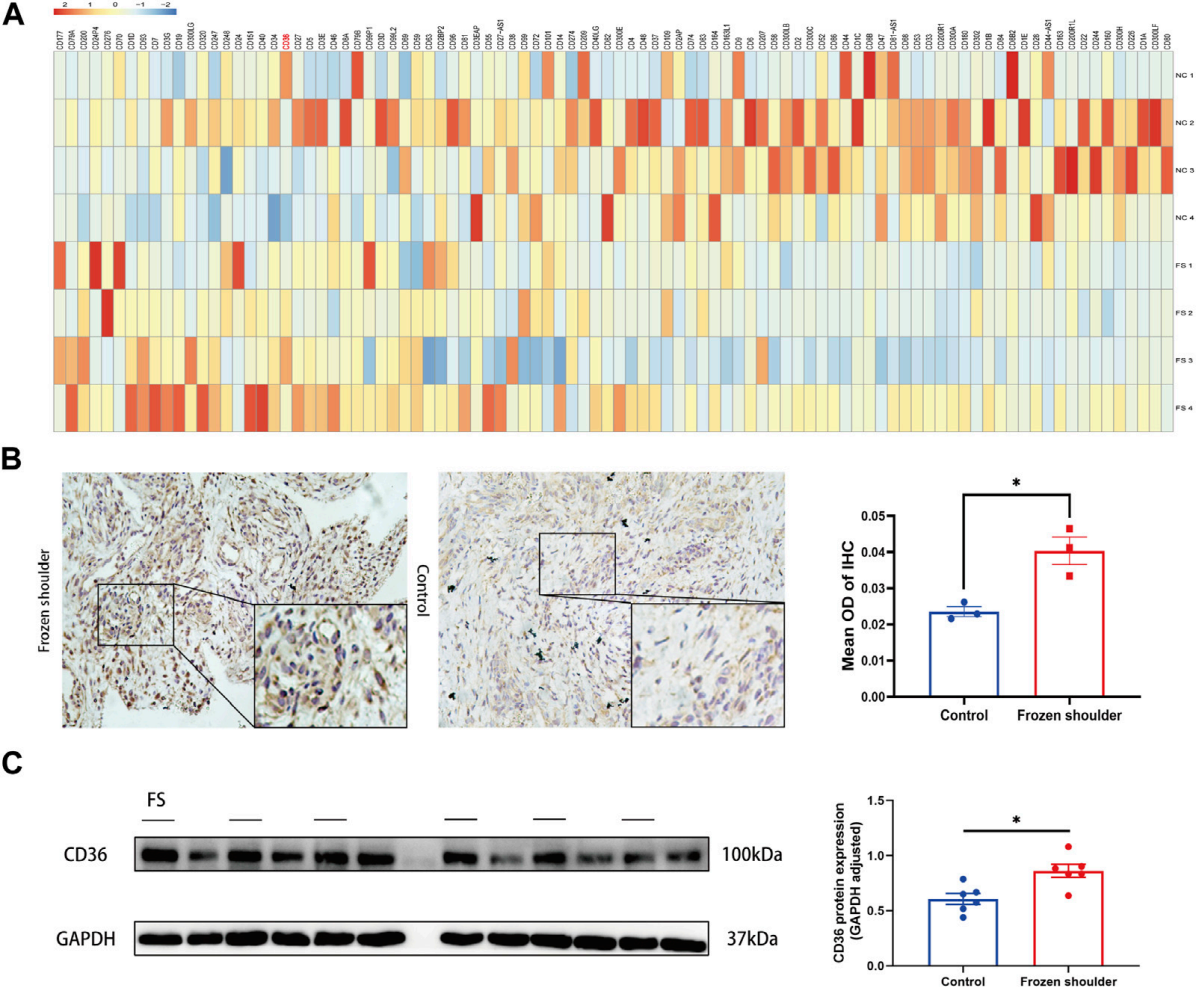
Different expression of *CD36* at both tissue and cellular level between FS and control group. **(A)** RNA-seq analysis suggested that CD36 was one of the most up-regulated molecules on surface of synovial fibroblasts. **(B)** Western blotting results showed that synovial fibroblasts in FS expressed more CD36 than that in the control group. Scale bar: 50 µm. **(C)** IHC results suggested that more CD36 was expressed in the synovium tissue derived from FS than that from the control group. *p < 0.05; **p < 0.01; ***p < 0.001.

Using RNA sequencing data from synovial tissue derived from 4 FS patients and 4 controls, we found that *CD36* was one of the most up-regulated genes encoding cell surface molecules of synovial fibroblasts (Figure 2A). In addition, the two most down-regulated microRNAs in FS compared to the control group, hsa-miR-2116-3p_L+1R-1 and hsa-miR-501-5p_R+2, both target *CD36* (Table 3), indicating that *CD36* RNA and protein levels may differ between FS and control groups and play a role in the development of FS.

**TABLE 3:** Differently expressed miRNA in NC and FS samples.

miRNA	Regulation in FS	log2 (FS/NC)	*p*-value (*t*-test)
hsa-miR-125a-3p_R-1	down	−0.30	0.000,440,812
hsa-miR-27b-3p	down	−0.57	0.003,894,912
mmu-miR-218-1-3p	down	−1.78	0.004,641,451
rno-miR-1843b-5p_L+1R-2_1ss19AG	down	−3.07	0.006,504,512
hsa-miR-224-5p_L-1R-2	down	−1.31	0.007,338,534
hsa-miR-3622a-3p_L-1R+1	up	inf	0.008,473,577
**hsa-miR-2116-3p_L+1R-1**	down	−1.93	0.012,183,296
eca-mir-8969-p3_1ss14 GT	down	−2.95	0.020,784,787
hsa-miR-15b-3p_R-1	down	−1.73	0.024,147,452
hsa-miR-16-2-3p_L+1R-1	down	−1.61	0.024,318,353
hsa-let-7i-5p	down	−0.60	0.024,500,581
PC-3p-51113_39	up	3.26	0.028,436,391
hsa-miR-450a-5p	down	−2.86	0.029,370,463
sha-miR-125a_R+2_2	up	1.82	0.030,383,164
sha-miR-125a_R+2_1	up	1.82	0.030,383,164
hsa-miR-125b-5p	up	0.59	0.031,412,209
**hsa-miR-501-5p_R+2**	down	−1.42	0.033,334,063
hsa-miR-154-5p	down	−1.45	0.033,908,402
bta-miR-199c_L-1R+1	down	−4.27	0.034,546,855
hsa-miR-151b_R+2	down	−1.09	0.037,277,433
bta-miR-2404	up	2.42	0.042,691,155
hsa-miR-664a-5p_R-2	down	−0.96	0.04,420,807
hsa-miR-107_R-2	down	−0.83	0.046,185,833
hsa-miR-369-5p_R-1	down	−1.75	0.046,591,148
hsa-miR-125a-5p_R-2	up	1.15	0.046,716,935
hsa-let-7i-3p	down	−0.95	0.046892
hsa-miR-214-5p	down	−1.11	0.047,246,093
hsa-miR-181a-3p	down	−1.25	0.049,249,894
ssc-miR-339_R+2	up	1.74	0.049,426,565

NC: normal control; FS: frozen shoulder.

To determine the expression of *CD36* at the tissue level, synovia from FS patients and control patients were collected under arthroscopy. By using IHC, we compared the expression of *CD36* in the synovium and found that CD36 was mainly present on the lining of blood vessels and the surface of synovial fibroblasts. In addition, *CD36* was significantly up-regulated in FS synovium, compared to controls (Figure 2B).

The significance of synovial fibroblast in the progression of pathologic fibrosis has already been established in pulmonary fibrosis, renal fibrosis, and liver fibrosis (Guyot et al., 2006; Sun et al., 2016; Tsoyi et al., 2018). We also previously demonstrated that synovial fibroblasts are the main effector cells in FS (Yang et al., 2022). To determine the expression of *CD36* at the cellular level, we performed Western blotting using protein from fibroblasts isolated and cultured from the synovium of both groups. In agreement with our IHC findings, our western blots showed that CD36 protein expression was considerably higher in synovial fibroblasts from FS patients compared to controls (Figure 2C). These findings demonstrate that FS is associated with a higher expression of *CD36* at the tissue and cell levels.

### 3.3 SaB inhibits inflammation response and fibrosis-related functions of synovial fibroblasts

**FIGURE 3:**
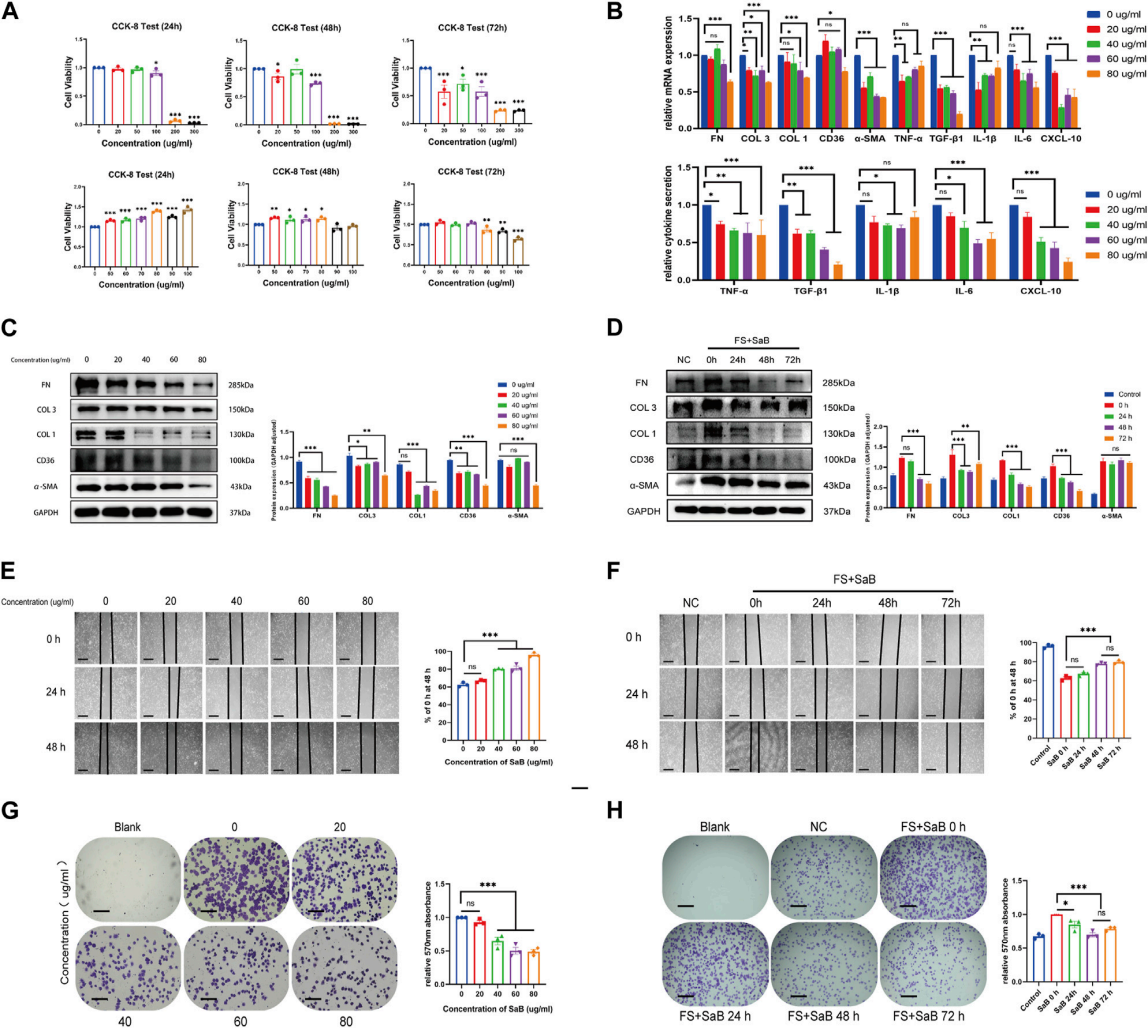
SaB inhibited inflammation response and fibrosis-related functions of synovial fibroblasts. **(A)** The CCK-8 result showed that with the increase of SaB concentration, the number of living synovial fibroblasts decreased gradually. SaB treatment less than 80 μg/mL have no statistically significant effect on the growth and proliferation of synovial fibroblast within 72 h. **(B)** qRT-PCR results showed a decreasing mRNA trend of inflammatory cytokines and fibrosis-related molecules with increasing SaB concentrations. ELISA results suggested that less inflammatory cytokines were secreted in the supernatant of synovial fibroblast after the administration of SaB. **(C)** Western blotting results showed that as the concentration of SaB increased, synovial fibroblast expressed less fibrosis-related proteins including COL 1, COL 3, FN, and *α*-SMA as well as CD36. **(D)** Synovial fibroblasts expressed less fibrosis-related molecules as the administration time of SaB increased within 72 h. Besides, administration time of 48 h showed no significantly different therapeutic effect than that of 72 h, or even better. **(E–H)** Cell adhesion test and Wound-healing suggested that SaB concentration of 80 μg/mL and administration time of 48 h may be the appropriate condition for inhibiting the fibrosis-related functions of synovial fibroblasts. Scale bar: 50 μm *p < 0.05; **p < 0.01; ***p < 0.001.

To test if CD36 offers an anti-fibrotic therapeutic target in FS, we exploited SaB as a specific inhibitor to interfere with the expression and function of *CD36* (Bao et al., 2012). We utilized a cell counting kit-8 test on fibroblasts cultured with a range of SaB concentrations (0, 20, 50, 100, 200, and 300 μg/mL) to establish its cytotoxicity. Increasing SaB concentrations were associated with a decrease in the number of viable synovial fibroblasts; the significant difference appeared at a concentration of 100 μg/mL. In response, we decided to use SaB at 0, 20, 40, 60, 80, and 100 μg/mL to identify the best concentration to antagonize CD36. Using CCK-8 test, we found that SaB <80 μg/mL had no significant effect on the growth and proliferation of synovial fibroblasts within 72 h (Figure 3A).

Consequently, different concentrations of SaB (0, 20, 40, 60, 80 μg/mL) were used in the synovial fibroblast culture medium to test its effects on inflammation response and fibrosis-related function by using RT-qPCR, ELISA, Western blotting, a cell adhesion test, and a wound-healing assay. In general, we found that the mRNA and protein expression of several fibrosis-related ECM and signals, including COL 1, COL 3, FN, and *α*-SMA, showed a decreasing trend with increasing SaB concentrations. Besides, the transcription and secretion of inflammatory cytokines (TNF-α, TGF-β1, IL-1β, IL-6 and CXCL-10) also decreased sharply after the stimulation of SaB. (Figures 3B,C,E,G). In short, SaB inhibited inflammation response and fibrosis-relevant processes in a dose-dependent manner. Additionally, higher concentrations of SaB corresponded with worse healing and adhesion function of synovial fibroblasts; the lowest extent of healing occurred at a concentration of 80 μg/mL (Figure 3E). We then tested SaB on synovial fibroblast over time (24, 48, and 72 h) and found that at 48 h there was greater inhibition than at 24 h. However, there was no significant difference at 72 h (Figures 3D,F,H). Thus we concluded that 80 μg/mL of SaB for 48 h may be beneficial to attenuating the synovial fibroblasts-induced fibrosis without exerting adverse effects on cell growth and proliferation. We also applied this regimen in subsequent experiments.

### 3.4 SaB inhibited pathologic fibrosis at the cellular level via CD36

We noted that as the concentration of SaB in the culture medium was increased, the expression of *CD36* in synovial fibroblasts was dramatically decreased at the mRNA and protein levels (Figures 3B, C); this finding is consistent with the trends identified in fibrosis-related molecules. However, we sought additional evidence to establish if SaB inhibited synovial fibroblast-induced fibrosis by acting via CD36.

**FIGURE 4:**
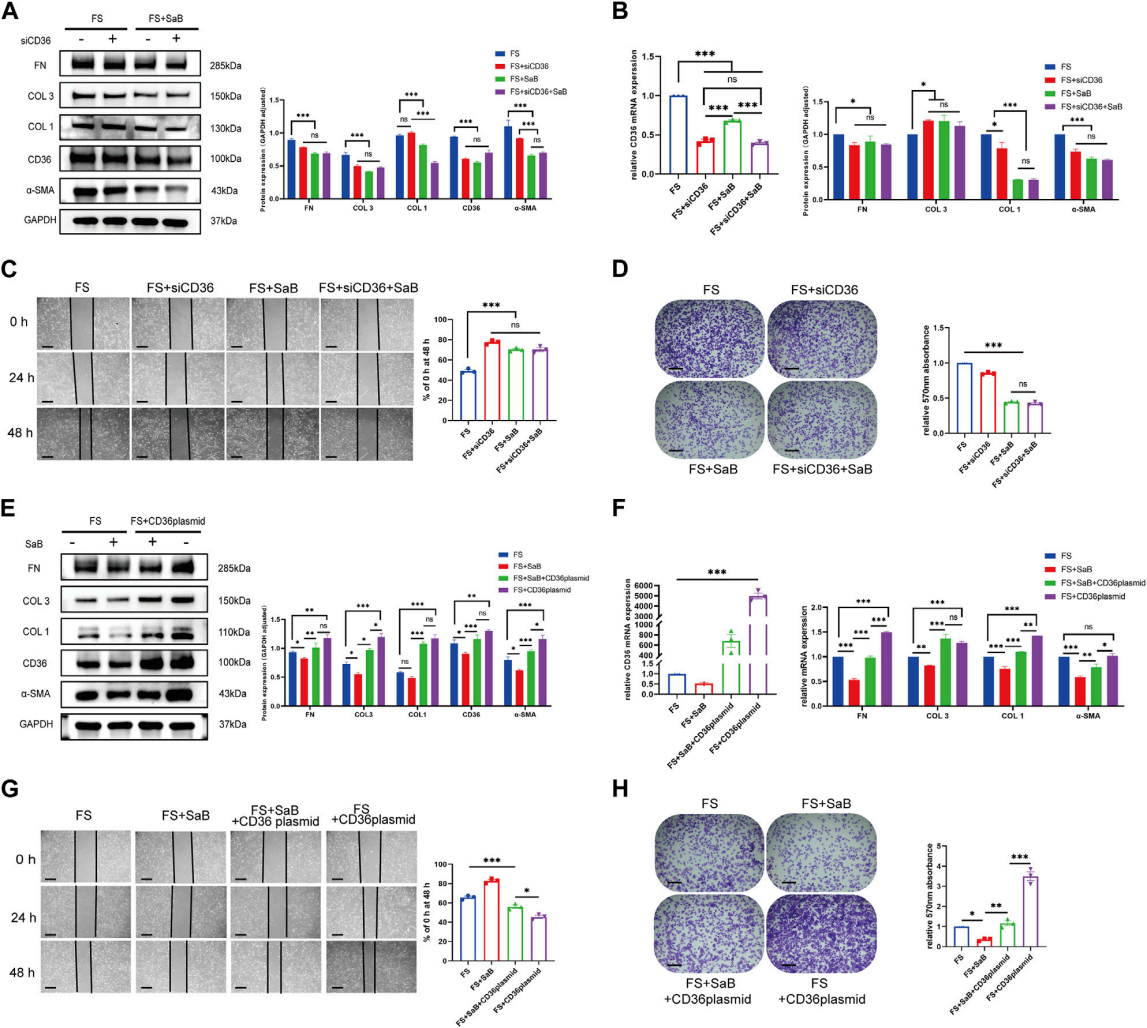
SaB inhibited pathologic fibrosis at the cellular level via CD36. **(A–D)** After knocking down *CD36*, fibrosis-related molecules (COL 1, COL 3, FN, and α-SMA) were down-regulated at both mRNA and protein levels in synovial fibroblast, the effect of which was similar to that seen after SaB treatment. Besides, the healing and adhesion abilities of synovial fibroblast were also impaired in a manner comparable to SaB treatment. Scale bar: 50 µm. **(E–H)** Synovial fibroblasts secreted more fibrosis-related molecules and showed considerably greater capacity for healing and adhesion after being transfected with *CD36* overexpression plasmid. When transfecting plasmid into synovial fibroblast cultured with SaB, the anti-fibrotic effect of which was partially reversed. Scale bar: 50 μm *p < 0.05; **p < 0.01; ***p < 0.001.

We tested the effect of *CD36* siRNA interference on fibroblasts generated from human synovium. After knocking down *CD36*, fibrosis-related molecules (COL 1, COL 3, FN, and *α*-SMA) were downregulated at the mRNA and protein levels in synovial fibroblasts (*p* < 0.05). We found that the associated decreases in the expression of COL 1, COL 3, FN, and *α*-SMA were similar to those seen following SaB treatment (Figures 4A, B). In addition, the healing and adhesion capacities of synovial fibroblasts were also impaired in a manner comparable to SaB treatment (Figures 4C, D). To further clarify the relationship between SaB and CD36, we transfected a *CD36* overexpression plasmid into synovial fibroblasts and found that, as a result, synovial fibroblasts secreted more fibrosis-related molecules and showed a considerably higher capacity for healing and adhesion. Interestingly, when transfecting plasmids into synovial fibroblast cultured with SaB, the anti-fibrotic effect was partially reversed (Figures 4E–H). Thus we believe that SaB inhibits pathologic fibrosis at the cellular level via CD36 while overexpression of *CD36* can attenuate or even reverse this effect.

### 3.5 Gene expression changes after *CD36* knockdown

**FIGURE 5:**
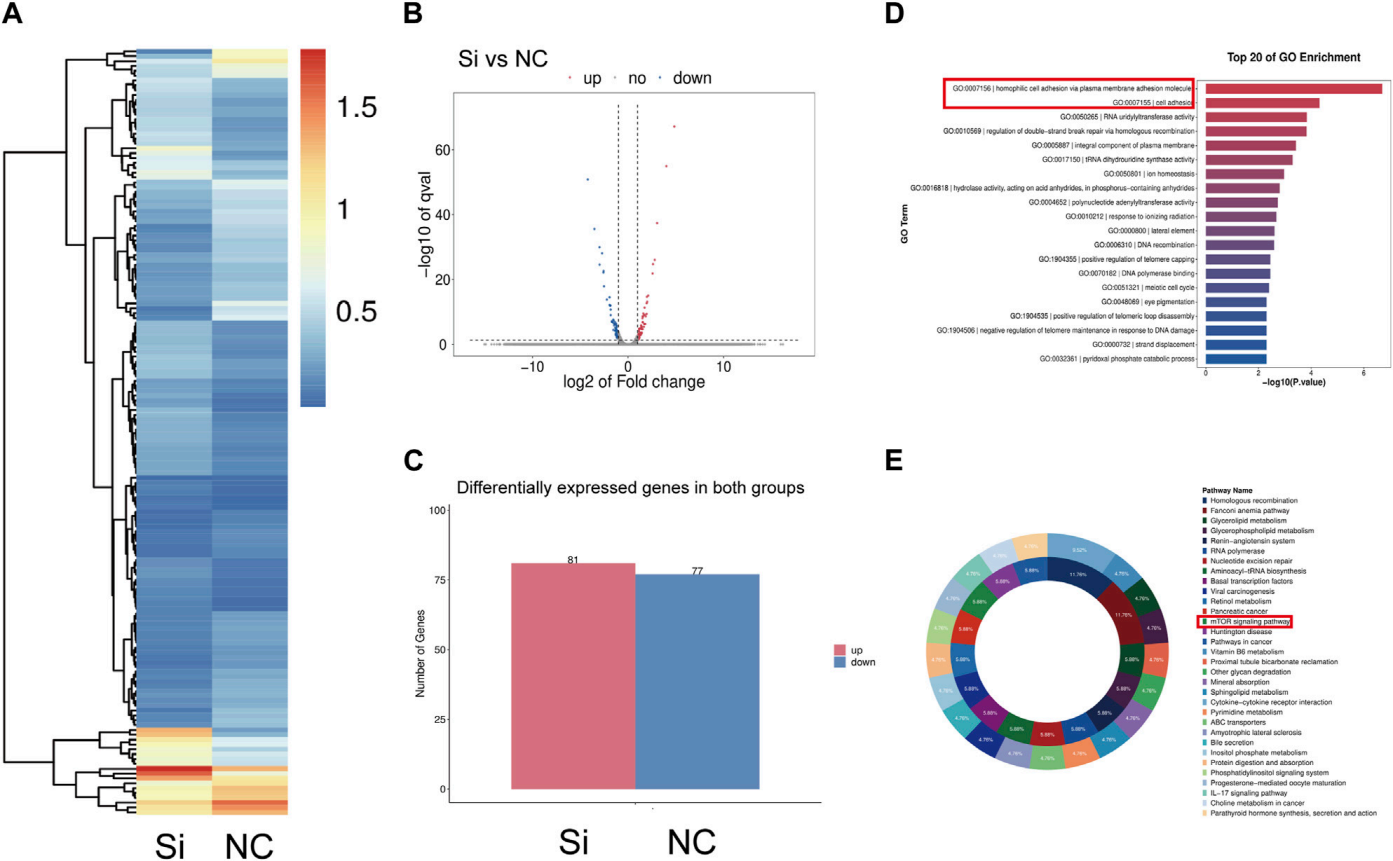
Gene expression change of synovium fibroblasts after *CD36* knockdown. **(A–C)** A total of 158 mRNA were differently expressed (Fold Change ≥2 and Adjusted *p*-value ≤0.001) between siRNA group and the control group, among which 81 were upregulated and 77 were down-regulated. **(D)** KEGG pathway analysis was performed to determine the pivotal signaling pathways within the differentially expressed genes. **(E)** GO analysis was performed to investigate the vital change of cell function after knocking down the *CD36* gene in synovial fibroblast.

To further clarify the relationship between CD36 and pathologic fibrosis, we first reviewed the RNA-seq data from FS patient synovial samples and those from control patients and found that the PI3K-Akt and MAPK pathways were two of the most upregulated in the FS group (Supplementary Figure S1). We consequently employed RNA-seq technology to examine the synovial fibroblast transcriptome from 6 individuals with FS; 3 of these underwent *CD36* knockdown interference using siRNA. We identified 158 differentially expressed genes (DEGs) (fold change ≥2 and adjusted *p*-value ≤0.001) between the siRNA and control groups; of these, 81 were upregulated and 77 were downregulated (Figures 5A–C). GO analysis was performed to investigate the functional implications of knocking down the *CD36* gene in synovial fibroblast. The Top-20 cell function change is shown in Figure 5D. Among all affected functions, homophilic cell adhesion via plasma membrane adhesion molecules (GO:0007156) and cell adhesion (GO:0007155) were the top-2 significant ones; this corresponded with our hypothesis that CD36 is crucial to cell adhesion, ECM deposition, and fibrosis. KEGG pathway analysis was also performed to determine the key signaling pathways related to the DEGs; all significant signaling pathways are shown in Figure 5E. Among these, the mTOR signaling pathway may play an important role between CD36 and synovial fibroblast-induced fibrosis, while the PI3K-Akt pathway was one of the main upstream signal sources of the mTOR signaling pathway. Of note, in our previous research, we demonstrated that IL-6 promotes synovial fibroblast-induced fibrosis by activating the PI3K-Akt signaling pathway (Yang et al., 2022). Thus we now speculate that the PI3K-Akt signaling pathway might be the main downstream pathway mediating the fibrosis-promoting signals from CD36.

### 3.6 CD36 promotes pathologic synovial fibroblast-induced fibrosis in FS through the PI3k-Akt pathway

**FIGURE 6:**
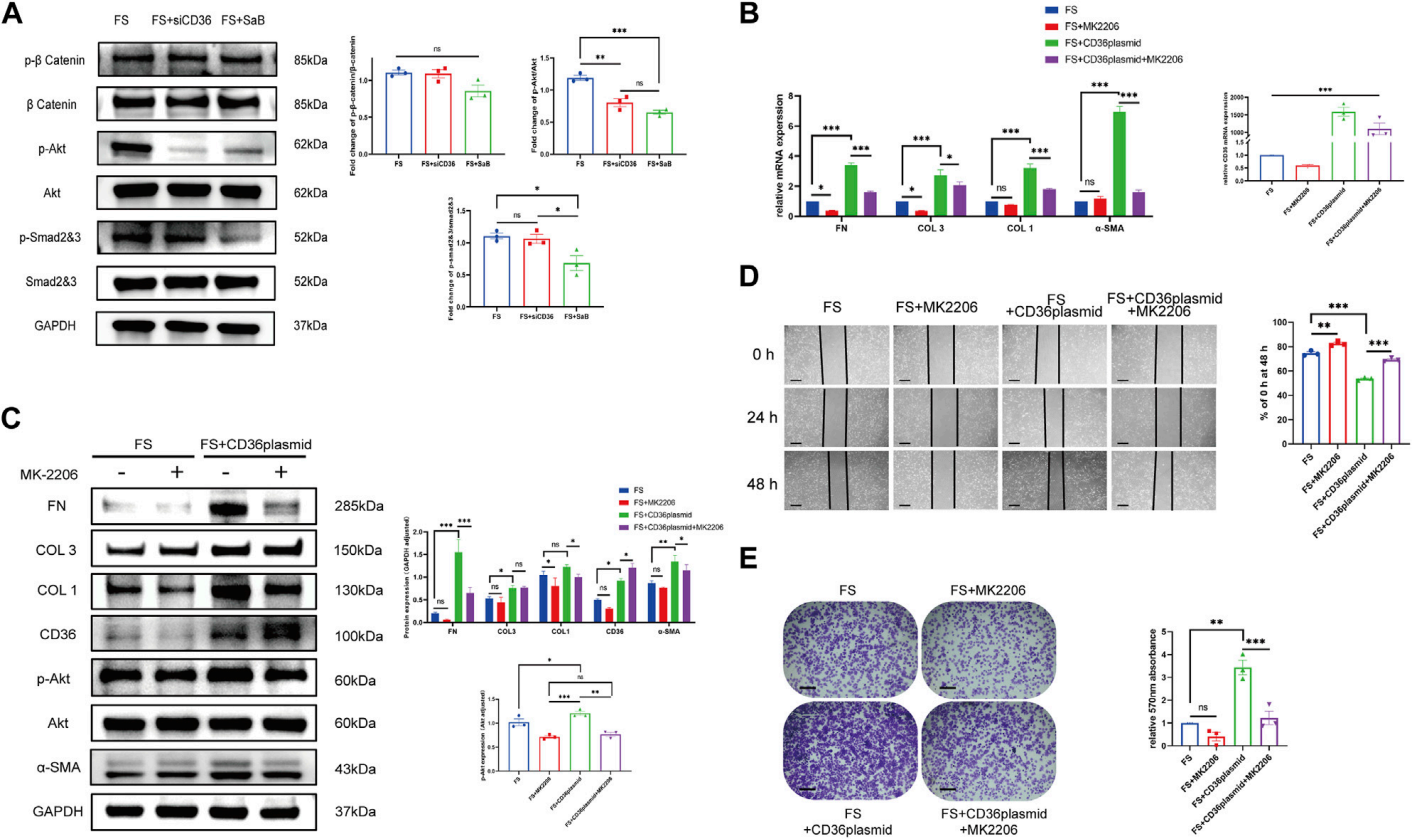
CD36 promotes pathologic synovial fibroblast-induced fibrosis in FS through the PI3k-Akt pathway. **(A)** The expression of p-Akt/Akt, as well as several key molecules in other classic fibrosis-related pathways including the TGF-*β*1/SMAD pathway and the WNT/β-catenin pathway, was examined in synovial fibroblasts treated with siRNA or SaB. **(B,C)** Western blotting and qRT-PCR results showed that COL 1, COL 3, FN and *α*-SMA along with Akt phosphorylation was significantly upregulated in FS synovial fibroblasts after the overexpression of *CD36*, and this trend was reversed by Akt inhibitor, MK2206. **(D,E)** Cell adhesion test and Wound-healing suggested that MK2206 inhibited the enhanced fibrosis-related functions in synovial fibroblasts caused by the overexpression of *CD36*. Scale bar: 50 μm *p < 0.05; **p < 0.01; ***p < 0.001.

To verify our hypothesis that CD36 promotes fibrosis via the PI3K-Akt signaling pathway, we performed Western blotting to test if the expression of key molecules in the PI3K-Akt pathway changed significantly after knocking down *CD36*. We also examined the expression of key molecules in several classic fibrosis-related pathways, including the TGF-β1/Smad pathway and Wnt/β-catenin pathway in synovial fibroblasts (Figure 6A). We found that, compared to controls, the expression of phosphorylated Akt decreased significantly in synovial fibroblasts after *CD36* siRNA interference or after culture with SaB. We also observed a non-significant reduction in the expression of Smad2/3 after knocking down *CD36* in synovial fibroblasts. As a result of these findings, we inferred that the PI3K-Akt signaling pathway plays a role downstream of CD36.

To better understand the role of the PI3K-Akt signaling pathway downstream of CD36 in frozen shoulder, we up-regulated the expression of *CD36* in synovial fibroblasts to investigate whether this affected the PI3K-Akt pathway. We added the specific Akt inhibitor MK2206 to synovial fibroblasts transfected with a *CD36* overexpression plasmid to block the PI3K-Akt pathway and assess if the development of pathologic fibrosis was blocked. Our RT-qPCR and Western blotting results suggested that the plasmid could effectively promote the expression of *CD36* in synovial fibroblasts of FS. We observed an increased expression of COL 1, COL 3, FN, and *α*-SMA along with PI3K and Akt phosphorylation in FS synovial fibroblasts after the overexpression of *CD36*; these trends were reversed by MK2206 (Figures 6B,C). Our wound healing assay and cell adhesion test supported these results (Figures 6D,E). These findings confirmed that CD36 promotes pathologic fibrosis of synovial fibroblasts in frozen shoulder through the PI3k-Akt pathway.

### 3.7 SaB blocks the progression of pathologic fibrosis of frozen shoulder *in vivo*

Finally, we targeted the CD36 molecule in an FS model induced in SD rats through the peritoneal injection of SaB for 3 weeks, to evaluate the direct effect of suppressing CD36 on the incidence and progression of FS.

#### 3.7.1 Gait analysis

**FIGURE 7:**
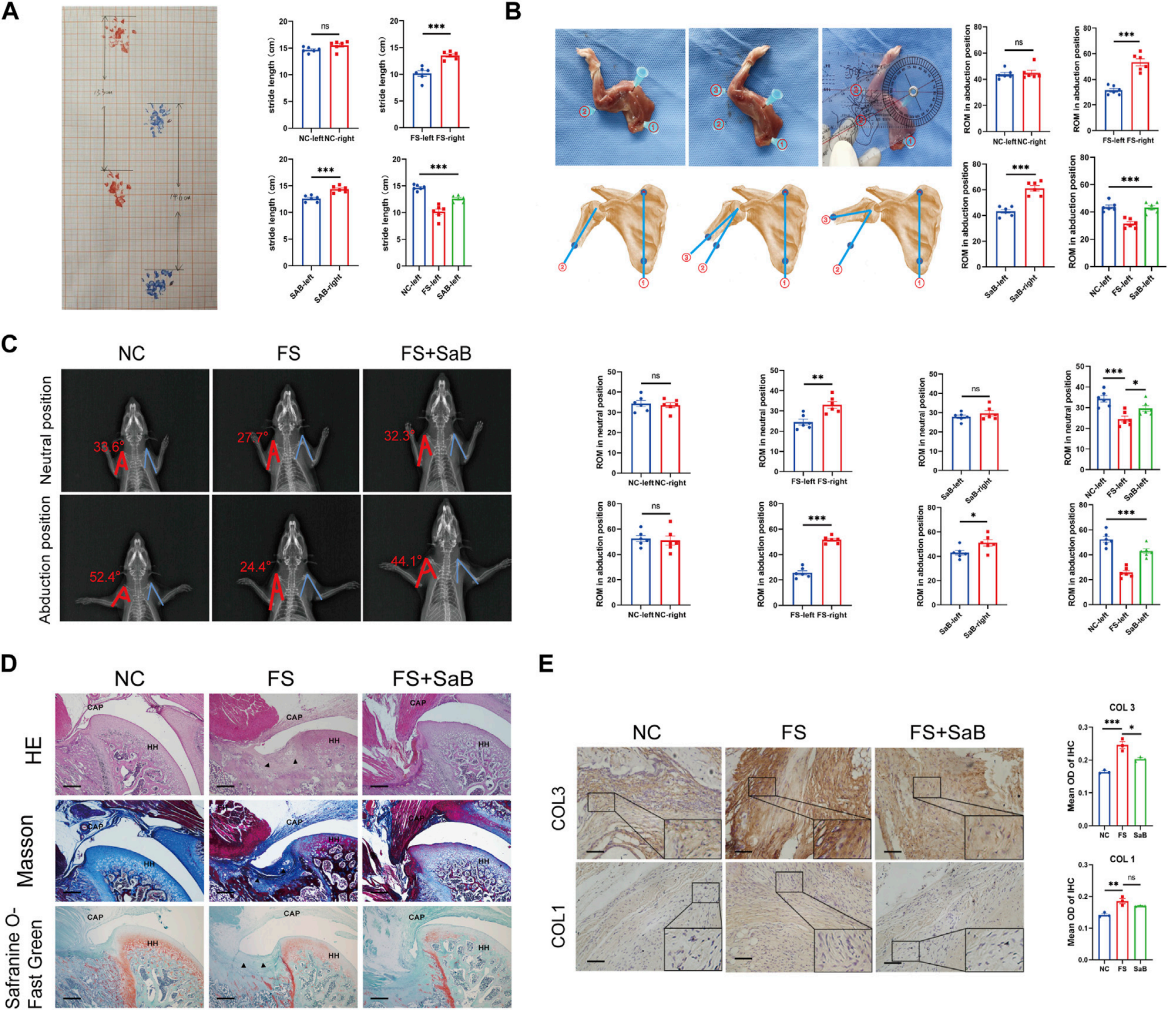
SaB blocks the progression of pathologic fibrosis of frozen shoulder *in vivo*. **(A)** Gait analysis by measuring the stride length on a grid paper. Stride length is defined as the longest distance between the front and rear paws. **(B)** Evaluation of the shoulder ROM. The angle between the scapular medial border (line 1) and humerus shaft (line 2 or 3) was measured, and the ROM was calculated by subtracting the angle in a maximally adducted position from the angle in a maximally abducted position. **(C)** The neutral and abduction positions’ X-ray films were collected using a flatbed scanner. Consider the axis of the humerus and the lateral edge of the scapula as the two sides of an angle with the humeral head’s center as the vertex. **(D)** The capsular areas were increased in the left immobilized shoulder in groups B and C, while group C showed a significantly smaller capsular area than that of group B. Besides, the amount of inflammatory cells observed in group C was significantly lower than that in group B, but higher than in group A, although not statistically significant. Black arrow: Deposition of collagen. HH: humeral head; CAP: capsule of joint. Scale bar: 100 µm. **(E)** Higher expression of COL 1 and COL 3 in capsule was observed in group B than the other 2 groups, while no significant difference was found between group A and C. Scale bar: 50 μm *p < 0.05; **p < 0.01; ***p < 0.001.

Gait analysis was performed after removing the left shoulder fixation. We found that Groups B and C had a significantly decreased stride length on the left compared to the right side (10.17 ± 1.29 cm vs. 13.53 ± 0.71 cm, 12.6 ± 0.59 cm vs. 14.4 ± 0.59 cm, respectively; *p* < 0.001), whereas group A showed no difference in stride length between sides (all *p* > 0.05). We found that group B showed a smaller stride length on the left than groups A and C (14.68 ± 0.5 cm in group A and 10.17 ± 1.29 mm in group B and 12.6 ± 0.59 cm in group C, *p* < 0.001). We also found a significant difference between groups A and C (*p* < 0.001) (Figure 7A). FS rats treated with SaB showed a significantly larger stride length compared to the plaster fixation and PBS injection only groups.

#### 3.7.2 ROM evaluation

To evaluate the shoulder ROM, we radiographically imaged the rats under general anesthesia. Significantly different abduction was found between the immobilized and control shoulders in groups B and C (25.7° ± 4.04°vs. 51.68° ± 2.83°, 42.95° ± 4.68°vs. 51.06° ± 6.47°, respectively; *p* < 0.05). There was no difference in ROM between both shoulders in group C. In between-group analyses of the left immobilized shoulders, group B had a significantly lower ROM than groups A and C (52.51° ± 5.84°in group A and 25.7° ± 4.04°in group B and 42.95° ± 4.68°in group C, respectively; *p* < 0.001); there was also a significant difference between groups A and C (*p* < 0.001) (Figure 7C).

The rats were then sacrificed. We measured the abduction angle on fresh cadaver specimens; a significant difference in shoulder ROM was observed between the left immobilized and right control shoulders in groups B and C (31.5° ± 3.45°vs. 53.33° ± 6.62°, 43.25° ± 3.74°vs. 61.17° ± 5.31°, respectively; *p* < 0.001). However, no similar difference was detected in group A (all *p* > 0.05). In the between-group analyses, group B showed significantly smaller ROM than groups A and C (43.75° ± 3.46°in group A and 31.5° ± 3.45°in group B and 43.25° ± 3.74°in group C, respectively; *p* < 0.001). However, there was no difference in the left shoulder ROM between groups A and C (*p* > 0.05) (Figure 7B). These findings indicate that FS rats treated with SaB had a significantly greater ROM compared to control animals treated with PBS.

#### 3.7.3 Histologic evaluation

We then performed H&E staining, Masson’s trichrome staining, and Safranin O-Fast Green staining to evaluate the local deposition of collagen fibers and the distribution of inflammatory cells around the shoulder capsule. We found that the capsular thickness was significantly increased in the left immobilized shoulders than that in the right ones in groups B and C. Similar capsular thickness were observed between both shoulders in Group A (Figure 7D). The between-group analyses of the right shoulders showed that group B had significantly larger capsular thickness than groups A and C, while group A showed a significantly smaller capsular thickness than group C (Figure 7D). In addition, in group C, we observed fewer inflammatory cells compared to group B, but slightly more than in group A.

Further, IHC of COL 1 and COL 3 was used to quantify the expression of collagen fibers. As shown in Figure 7E, group B had significantly higher capsular COL 1 and COL 3 than the other two groups, but there were no significant differences between groups A and C. Based on these findings, SaB can help prevent and treat pathologic fibrosis of FS in an *in vivo* rodent model.

## 4 Discussion

**FIGURE 8:**
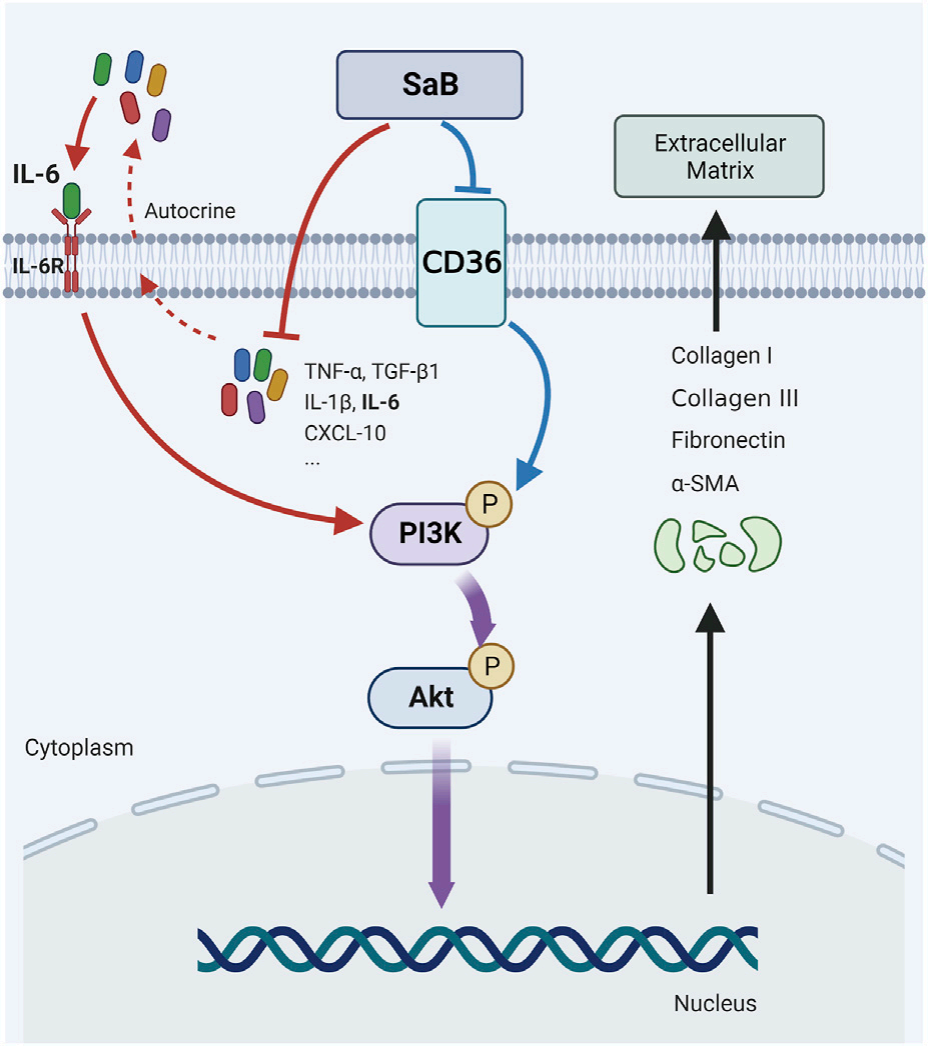
*CD36* were upregulated in synovial fibroblasts of FS and promote pathologic fibrosis in FS through the PI3k-Akt signaling pathway; SaB inhibited inflammation response and CD36-mediated pathologic fibrosis of FS. Created by Biorender.com.

In the current study, we found that *CD36* was significantly upregulated in synovium derived from the FS group at both tissue and cellular levels. This indicates that CD36 might be associated with the occurrence and development of FS. As an inhibitor of CD36, we used SaB against synovial fibroblasts to impair the inflammation response and fibrosis-related functions of synovial fibroblasts. We found that the anti-fibrotic effect of SaB could be blocked by overexpressing CD36, indicating that SaB inhibited pathologic fibrosis at the cellular level by interacting with CD36. Bioinformatics analysis of RNA-seq data indicated that the cell adhesion ability of synovial fibroblast was impaired significantly after knocking down *CD36*, and the PI3K-Akt signaling pathway might play a vital role in the fibrotic process of FS; we also showed this at the protein level. In addition, the expression of COL 1, COL 3, FN, and *α*-SMA increased alongside phosphorylated Akt in synovial fibroblasts after *CD36* was upregulated by using an overexpression plasmid; this trend was reversed by using the specific inhibitor of phosphorylated Akt, MK2206. These findings confirmed that CD36 promotes synovial fibroblast-induced pathological fibrosis in FS by activating the PI3k-Akt pathway. Finally, we used a peritoneal injection of SaB to target CD36 in FS model SD rats to verify its therapeutic effect on pathologic fibrosis *in vivo*. Animals treated with SaB showed significantly larger stride length, greater ROM, and less collagen fiber deposition than rats in the FS model group. Therefore, SaB inhibited CD36-mediated pathologic fibrosis in *vitro* and *in vivo* models (Figure 8).

FS is characterized by pain and ROM limitations. Inflammation and fibrosis are the key targets of FS research (Tamai et al., 2014). It has been shown that immune cells and inflammatory cytokines are present in FS synovium (Rodeo et al., 1997; Kabbabe et al., 2010) and increasing evidence points to inflammation as the precursor and initiator of fibrosis, while pathologic fibrosis remains the leading cause of limited ROM in FS (Hagiwara et al., 2012; Tamai et al., 2014). Pathologic fibrosis is mainly characterized by the excessive deposition of ECM, especially various collagen proteins (Bunker et al., 2000; Parola and Pinzani, 2019). Fibroblasts are the key effector cells in fibrosis, while synovial fibroblasts are the primary constituents of synovium (Hand et al., 2007). It has been shown that synovial fibroblasts are the main effector cells in synovial fibrosis in various diseases such as osteoarthritis, rheumatoid arthritis, and joint contractures (Remst et al., 2015; Jiang et al., 2018; Maglaviceanu et al., 2021). Previously, we demonstrated that synovial fibroblasts from FS have higher levels of ECM expression compared to controls, suggesting that these cells are the main effectors of pathologic fibrosis in FS (Yang et al., 2022). Based on our RNA sequencing analysis of synovial tissue, we noticed that the expression of miRNA targeting *CD36* mRNA was significantly lower in the FS group compared to the healthy control group. Thus we propose that CD36 participates in fibrosis.

As a scavenger receptor, CD36 participates in lipid transport and innate immunity (Silverstein and Febbraio, 2009). Interestingly, by targeting liver HSC cells in nonalcoholic fatty liver disease, previous studies suggested that CD36 is related to liver cirrhosis and might promote fibrosis (Lin et al., 2019). In additional studies, CD36 was also shown to play a role in skin scarring, pulmonary fibrosis, and renal fibrosis (Chen et al., 2009; Souza et al., 2016; Liu et al., 2021). In this study, we demonstrated that CD36 was highly expressed in FS at both tissue and cell levels. To further explore the regulatory effect of CD36 on fibrosis, we used SaB as a CD36 inhibitor (Bao et al., 2012). SaB dramatically decreased the expression of TNF-α, TGF-β1, IL-1β, IL-6, CXCL-10 and fibrosis-related proteins in synovial fibroblasts; this was accompanied by a reduction in healing and adhesion ability. Generally, SaB shows the dual potential of anti-inflammation and anti-fibrosis. In the future, SaB may be used externally in the form of transdermal patch or injected with hydrogel to treat FS.

FS progresses through three consecutive stages (Neviaser and Neviaser, 2011). Inflammatory cells and inflammatory factors play different roles in each period. In the early stage, acute inflammatory synovial reaction and rare inflammatory cell infiltration could be characterized on biopsy. While in late stage, chronic and unresolved inflammation are more common, which is associated with increased vascularity, proliferation of fibroblast, synovium thickening and ECM deposition (Kingston et al., 2018). Based on this, the role of TGF-β1 became the main emphasis of cytokine studies in FS. TGF-β1 is highly expressed in FS synovium and can induce cellular fibrotic responses, including ECM production, fibroblast proliferation and myofibroblast differentiation (Kraal et al., 2020). Other inflammatory mediators, including TNF, IL-1, IL-6, IL-10, M-CSF, PDGF and CXCL, are also dysregulated in FS and may drive inflammatory responses and pathologic fibrosis. Fibroblasts derived from FS capsule expressed elevated levels of pro-inflammatory cytokines in comparison with the levels produced by those from healthy controls (Bunker et al., 2000; Akbar et al., 2019). Although it is generally believed that inflammation may lead to the final manifestation of fibrosis, the inner relationship between inflammation and fibrosis is still not clear. In the exploration of the interaction of inflammatory factors, we found that NF-κB, as the key hub of inflammatory pathway, may be related to the process of fibrosis (Supplementary Figure S4). Besides, the role of synovial macrophages in the construction of inflammatory microenvironment and local pathological fibrosis of FS is also in mist. Further research is urgently needed in this direction.

Previously, we have demonstrated that IL-6 were upregulated in synovial fibroblasts of FS and promoted the FS fibrosis through PI3K-Akt signaling pathway (Yang et al., 2022). SaB show its ability to downregulate the expression of IL-6 and collagen. Thus we believe that CD36 antagonism using SaB can block synovial fibroblast-related fibrosis. However, considering the complex function of SaB, which include anti-inflammatory, anti-oxidant, and anti-fibrosis effects, we cannot completely rule out that SaB may interferes with fibrosis through CD36-dependent and CD36-independent ways (Xiao et al., 2020). Therefore, we compared the anti-fibrosis effect between synovial fibroblasts treated with SaB and synovial fibroblast with a *CD36* knock down, but found no significant difference between these conditions. In addition, the effect of SaB was reversed after the upregulation of *CD36*. Therefore, we concluded that SaB inhibited pathologic fibrosis in synovial fibroblasts via CD36, which is consistent with the findings of an earlier study focusing on scar hyperplasia (Liu et al., 2021).

To explore the underlying mechanism of CD36-mediated fibrosis, we performed RNA-seq on mRNA isolated from synovial fibroblasts with normal and reduced *CD36* expression. In our GO analysis, we found significant changes in cell adhesion ability while our KEGG analysis suggested significant differences in molecules involved in the mTOR pathway. Considering that the primary signal transmitting through the mTOR pathway arises from the PI3k-Akt signaling pathway, we speculated that the latter pathway mediates the signal of fibrosis downstream of CD36, which echoed our previous finding that IL-6 mediates fibrosis through the PI3K-Akt pathway (Yang et al., 2022). The PI3K-Akt pathway is a well-studied intracellular signal transduction pathway that responds to extracellular signals and promotes metabolism, proliferation, cell survival, growth, and angiogenesis (Ersahin et al., 2015). It has also been reported that the PI3K-Akt pathway is involved in various fibrotic processes (Wu et al., 2017; Qin et al., 2021). Therefore, we regulated the expression of *CD36* and interfered with phosphorylated Akt using MK2206 to verify whether the PI3K-Akt pathway affects the fibrosis process downstream of CD36. As shown in Figure 6, fibrosis-related proteins and PI3K-Akt-associated molecules increased synchronously after a simple overexpression of *CD36*, and the capacity of synovial fibroblasts for adhesion and healing was also improved. These trends were reversed by MK2206. In general, we found that CD36 promotes fibrosis through the PI3K-Akt pathway, while SaB inhibits the PI3K-Akt pathway-mediated fibrosis via CD36, suggesting that SaB may be a potential therapeutic for FS.

To further test the effect of SaB on FS, we established an *in vivo* rat FS model and administered SaB by intraperitoneal injection. Based on research published by Kim et al., we expected marked ROM limitation and local fiber deposition after 3 weeks of shoulder fixation (Kim et al., 2016). We noticed that the stride length and the ROM of the shoulder joint in the SaB group were significantly greater compared to the FS model group, but worse than the control group. In addition, our histological analysis suggested that the thickness of the shoulder capsule was decreased in the SaB group, and the expression of collagen fiber was significantly reduced when compared to the FS model group. Thus, based on our *in vitro* and *in vivo* work, we concluded that SaB shows considerable anti-fibrotic effects on FS.

There are some limitations in this study. First, as a classic traditional Chinese medicine, *Salviae miltiorrhizae* has very complex effects that include anti-atherosclerosis, anti-inflammation, anti-oxidation, and others. Therefore, we suspect that while the therapeutic effect of SaB on FS is CD36-dependent, it may also have other therapeutically useful effects such as anti-inflammation and anti-oxidation. Second, while fibroblasts are the main effector cells of fibrosis, they are not the only cells involved in this pathology. Although we demonstrated that the regulation of *CD36* and its downstream pathway in synovial fibroblast inhibits pathologic fibrosis, the interaction between fibroblasts and other cells such as macrophages or other cell products such as cytokines remains to be elucidated. Finally, the upstream genes that regulate the expression of *CD36* along with its intrinsic mechanisms were not interrogated in this study; these need to be further explored in future research.

## 5 Conclusion

We provide evidence that *CD36* is up-regulated in synovial fibroblasts of FS, promotes pathologic fibrosis through the PI3k-Akt signaling pathway. SaB attenuates inflammation and inhibits CD36-mediated pathologic fibrosis in *vitro* and *in vivo* models.

## Data Availability

The original contributions presented in the study are included in the article/Supplementary Material, further inquiries can be directed to the corresponding authors. The RNA-seq data presented in the study are deposited in the GEO repository, accession number GSE238054.

## Abbreviations

FS, Frozen shoulder; ROM, Range of motion; SaB, Salvianolic acid B; ECM, Extracellular matrix; CD36, Cluster of differentiation 36; H&E, Hematoxylin and eosin; IHC, Immunohistochemistry.
