# Supplementary figures and images for: Salvianolic acid B attenuates inflammation and prevent pathologic fibrosis by inhibiting CD36-mediated activation of the PI3K-Akt signaling pathway in frozen shoulder
Source: Front Pharmacol. 2023 Aug 1;14:1230174. doi: 10.3389/fphar.2023.1230174 (PMC10427508; doi:10.3389/fphar.2023.1230174)

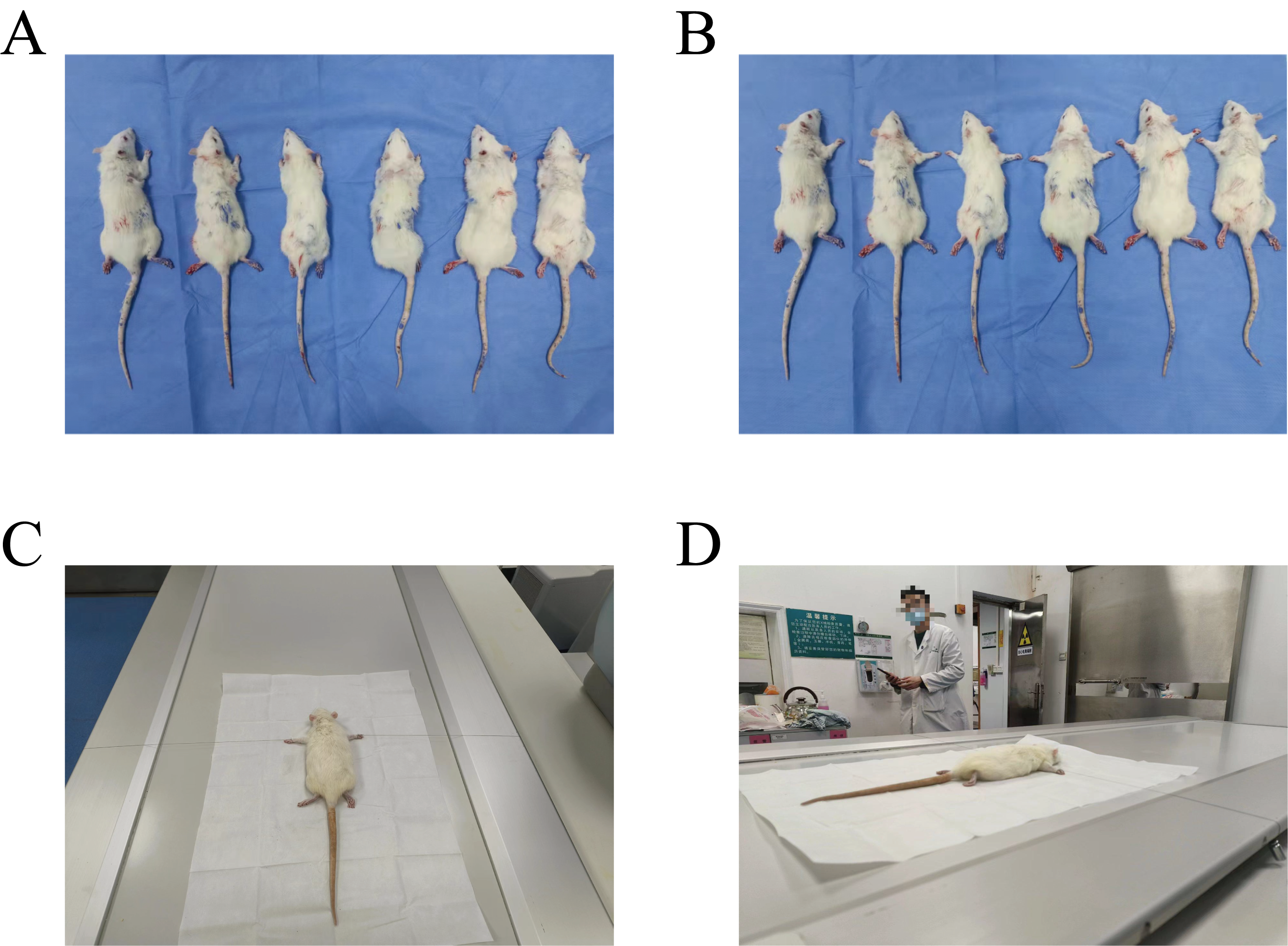

Supplement: Supplementary file 1 [file Image3.TIF]

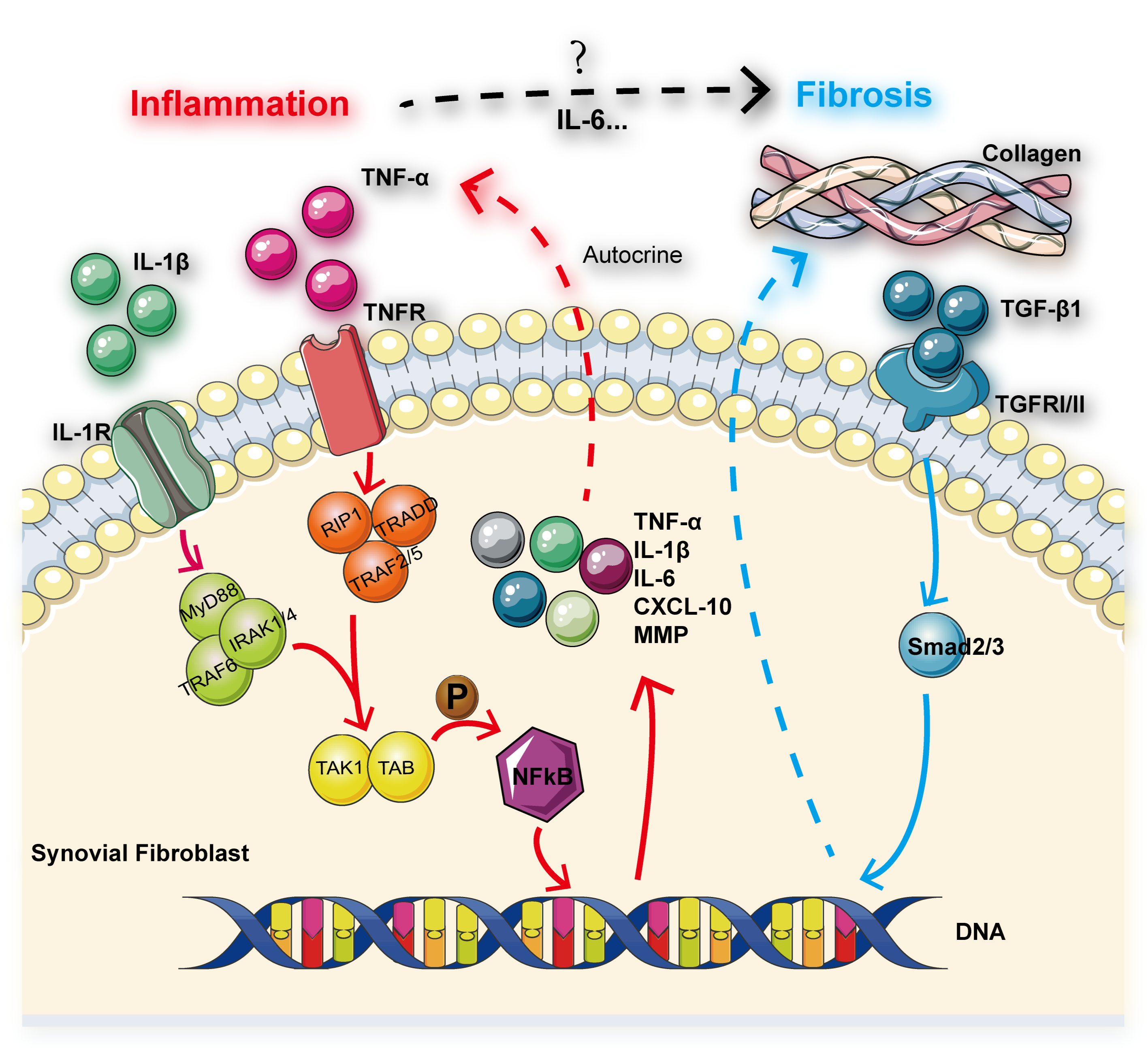

Supplement: Supplementary file 2 [file Image4.TIF]

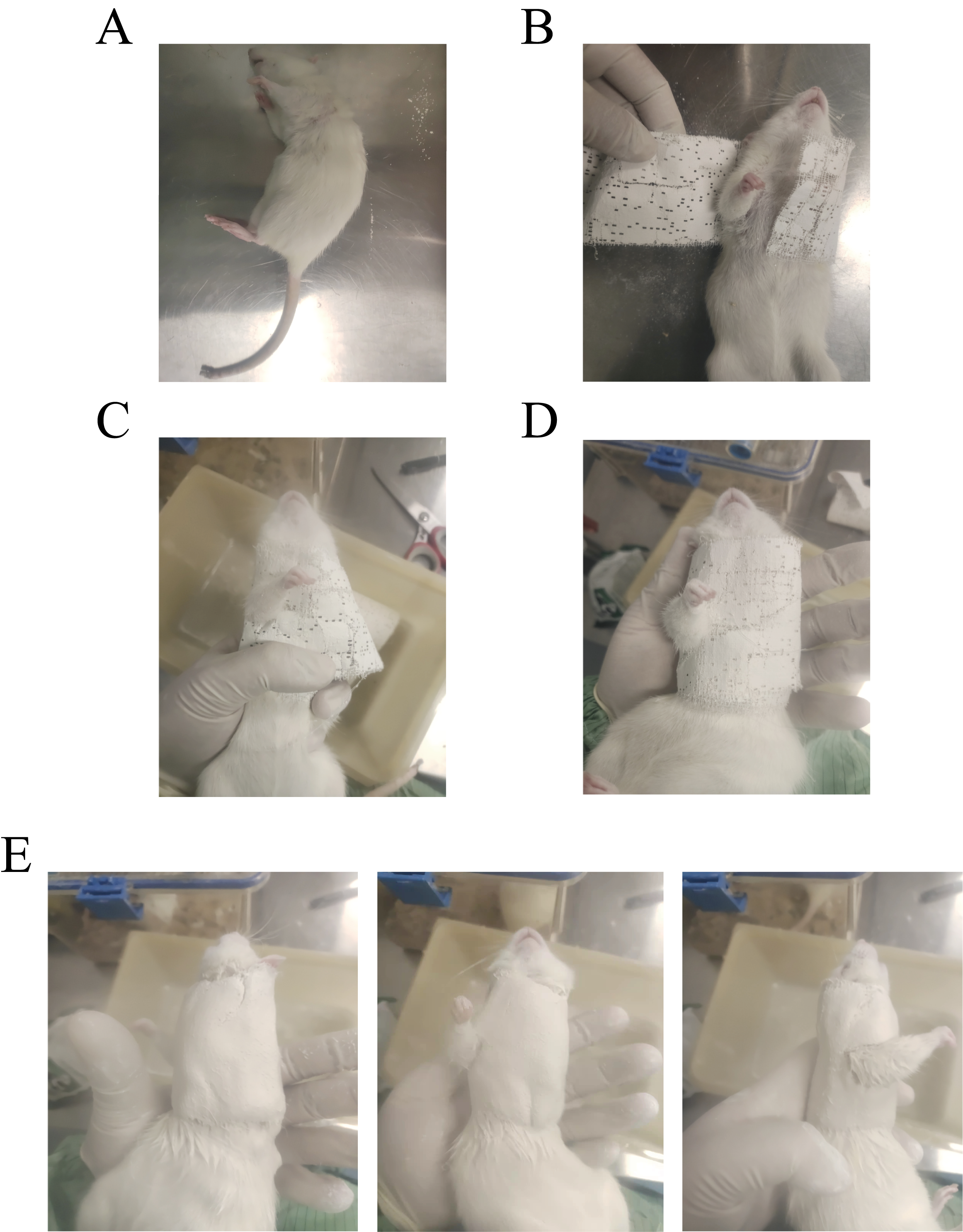

Supplement: Supplementary file 3 [file Image2.TIF]

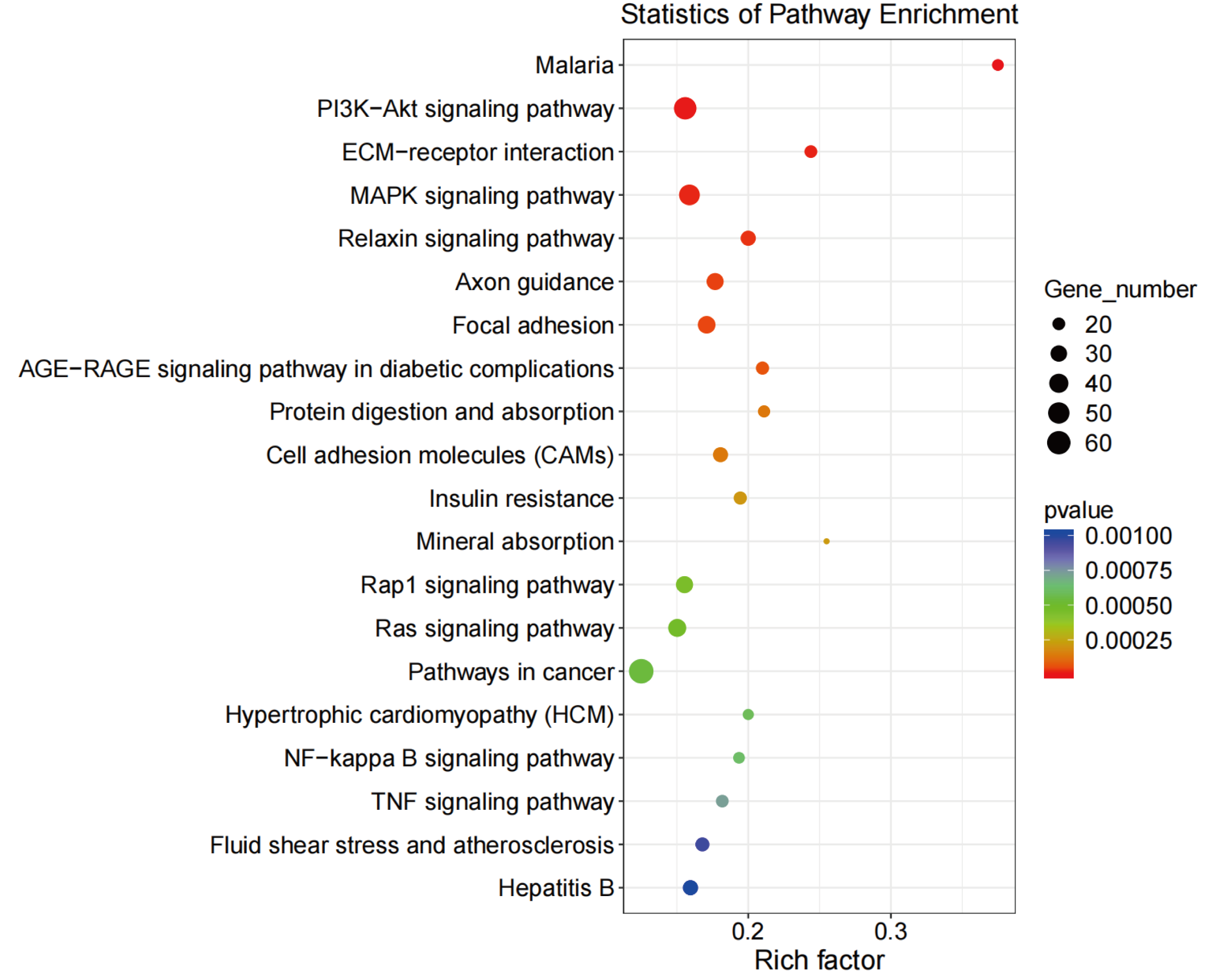

Supplement: Supplementary file 4 [file Image1.TIF]
